# India Rubber

**Published:** 1862-07

**Authors:** 


INDIA RUBBER.—In the report of the proceedings of the Polytechnic Association of the American Institute, in a cotemporary, it is stated that Professor Seely made the following remarks in relation to this substance: "When India rubber was first introduced, we thought that it would answer for all purposes. I proposed, myself, to use it as a steam engine in the form of a bellows. But we soon discovered that its properties were destroyed by the action of many substances, spirits of turpentine, acids, etc., and they were altered by changes in temperature and by the action of the air. I procured a very pure sample, as white as milk, but the surface soon became dark, and its tenacity was destroyed. It could be scraped off with a knife; it absorbed oxygen, and was changed into a resin, like some other hydrocarbons—spirits of turpentine, for instance, which became first oil of turpentine and then solid resin.

"When the art of vulcanizing was discovered, it was thought at first that all of the difficulties were overcome. But experience has shown that vulcanized rubber is acted upon by all the agents which affect the crude material, only in less degree, or more slowly. It becomes stiff in the cold, it is softened by heat, is dissolved slowly in spirits of turpentine and benzole, is spoiled if kept any considerable time in contact with grease, and is changed into resin by absorbing oxygen from the atmosphere. But all of these changes go on much more slowly in vulcanized rubber than in the crude material.

"There is another difficulty with vulcanized rubber,—that is, the adulteration. Perhaps there is no other manufacture in which adulteration is so systematically and extensively practiced as in that of India rubber. Some of the articles in market do not contain 10 per cent. of rubber. Notwithstanding these depreciatory remarks, I regard India rubber as a very valuable substance. I should almost agree with Liebig in the opinion that the four most valuable materials for the chemist—naming them in the order of their importance—are glass, platinum, cork, and India rubber."

